# Current Progress of Avian Vaccines Against West Nile Virus

**DOI:** 10.3390/vaccines7040126

**Published:** 2019-09-23

**Authors:** Nereida Jiménez de Oya, Estela Escribano-Romero, Ana-Belén Blázquez, Miguel A. Martín-Acebes, Juan-Carlos Saiz

**Affiliations:** Department of Biotechnology, National Agricultural and Food Research and Technology Institute (INIA), 28040 Madrid, Spain; jdeoya@inia.es (N.J.d.O.); eescribano@inia.es (E.E.-R.); blazquez@inia.es (A.-B.B.); martin.mangel@inia.es (M.A.M.-A.)

**Keywords:** birds, vaccines, West Nile virus, flavivirus, herd immunity

## Abstract

Birds are the main natural host of West Nile virus (WNV), the worldwide most distributed mosquito-borne flavivirus, but humans and equids can also be sporadic hosts. Many avian species have been reported as susceptible to WNV, particularly corvids. In the case that clinical disease develops in birds, this is due to virus invasion of different organs: liver, spleen, kidney, heart, and mainly the central nervous system, which can lead to death 24–48 h later. Nowadays, vaccines have only been licensed for use in equids; thus, the availability of avian vaccines would benefit bird populations, both domestic and wild ones. Such vaccines could be used in endangered species housed in rehabilitation and wildlife reserves, and in animals located at zoos and other recreational installations, but also in farm birds, and in those that are grown for hunting and restocking activities. Even more, controlling WNV infection in birds can also be useful to prevent its spread and limit outbreaks. So far, different commercial and experimental vaccines (inactivated, attenuated, and recombinant viruses, and subunits and DNA-based candidates) have been evaluated, with various regimens, both in domestic and wild avian species. However, there are still disadvantages that must be overcome before avian vaccination can be implemented, such as its cost-effectiveness for domestic birds since in many species the pathogenicity is low or zero, or the viability of being able to achieve collective immunity in wild birds in freedom. Here, a comprehensive review of what has been done until now in the field of avian vaccines against WNV is presented and discussed.

## 1. Introduction

Currently, the ecology of many pathogens is changing because of climate warming that is driving vector colonization of new geographical niches. This fact, together with human behavior and global trade, puts human and animal health at risk. An example is the (re)emergence of West Nile virus (WNV) that nowadays is the most worldwide distributed mosquito-borne flavivirus [1,2]. Since the introduction of a lineage 1 WNV strain in the US in 1999, the virus quickly spread, causing hundreds of deaths in humans and horses and a very high avian mortality [1,2]. More recently, the strains of lineage 2 colonized and spread throughout Europe, leading to outbreaks among wild birds [3] and being responsible for up to 1.875 human cases, including 115 deaths in 2018 [4].

WNV is a small (about 50 nm of diameter), spherical, enveloped flavivirus (*Flaviviridae* family) whose genome consists of a single-stranded RNA molecule of positive polarity that encodes three structural proteins and seven non-structural proteins [1]. Up to nine distinct genetic lineages of WNV have been described, with lineage 1 and 2 being the most distributed worldwide, although only a single serotype is recognized [1,5].

Birds are the main natural host of West Nile virus, though humans and equids can also be sporadically infected [1,2]. Hundreds of avian species have been reported as susceptible to WNV, particularly corvids (Corvidae), which can develop high levels of viremia [6,7], and are notable virus amplifiers [7,8,9], being, thus, important actors in the epidemiology of the virus [10,11,12,13,14]. Both domestic and wild avian species are susceptible to WNV infection and, in some cases, develop a WNV-associated disease that can lead to high mortality, as occurred during the US outbreak where crow populations declined alarmingly [14,15,16,17].

Currently, there is no antiviral therapy against WNV, and the licensed vaccines are only for use in equids [1,18,19]. The availability of avian vaccines would benefit bird populations, both domestic (like farm birds and those grown for restocking and hunting activities) and wild ones (mainly endangered species housed in rehabilitation and wildlife reserves, and birds located at recreational facilities like zoos). Avian vaccination may also help to prevent outbreaks and spread, mainly if herd immunity can be induced. Here, a comprehensive review of our current knowledge, about experimental avian vaccination with different candidates (inactivated, attenuated, and recombinant viruses, and subunit and DNA-based vaccines) in domestic and wild birds, is presented.

## 2. WNV Biology

### 2.1. Genome Organization

**Figure 1:**
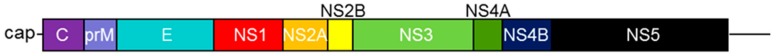
Genome organization. Schematic representation of the WNV (West Nile virus) genome. See text for details.

The genome of WNV is composed of a single-stranded positive-sense RNA (ssRNA(+)) of about 11 kb in length (Figure 1) [1]. It contains a 5’-cap structure (m(7)GpppAm) that is methylated at the guanine N-7 and the ribose 2’-OH positions of the first transcribed adenine [20] but lacks a 3’ polyA tail. The single open reading frame (ORF) is flanked by two untranslated regions (UTRs) with important functions for viral replication [21]. Remarkably, the 3’ UTR is a key determinant of WNV virulence, which makes it attractive for vaccine design [22]. The ORF is translated into a polyprotein that is co-translational and post-translationally cleaved by viral and cellular proteases. The structural capsid (C) protein is involved in the nucleocapsid formation by association with the genomic RNA, the M is produced by cleavage of the prM, and the E is involved in receptor binding, viral entry, and membrane fusion [23]. The non-structural NS2B is the membrane anchor and the co-activator of the NS3 viral serine protease. The NS1 is secreted and has been related to replication, virulence, immunomodulation, and pathogenesis [24]. The NS5 exhibits the methyltransferase activity required for capping of viral RNA and is also the RNA-dependent RNA polymerase in charge of genome replication [21]. Replication of WNV is associated with intracellular membranes of the Endoplasmic Reticulum (ER) [25]. Accordingly, NS2A, NS2B, NS4A, and NS4B are multipass transmembrane proteins. The ER is the place for viral replication and particle biogenesis. The newly assembled immature particles are produced by budding into the lumen of this organelle and traffic across the secretory pathway. Viral particles maturate towards infectious virions by proteolytic processing of the prM to render the M protein. This cleavage takes place inside the *trans*-Golgi network and is catalyzed by the cellular protease furin [23]. Maturation converts the spiky immature particles [26] into smooth mature virions [27] that are released from the cell by exocytosis. 

### 2.2. Molecular Classification and Phylogeny

**Figure 2:**
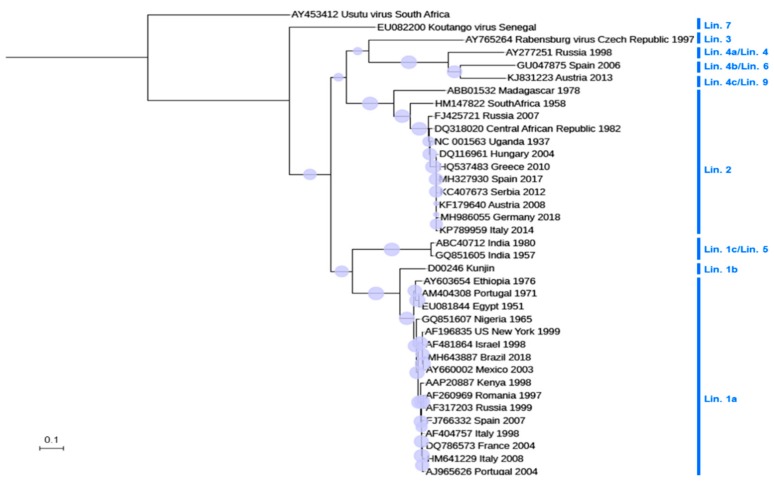
Phylogram, showing the relationships between the WNV strains. The tree is based on the complete nucleotide sequence of NS5 (except for HU2925/06 and MH327930). Multiple alignment was performed using MUSCLE [32], and a maximum likelihood tree was built using W-IQ-TREE [33]. The tree was visualized with iTOL [34]. Usutu virus was included as an outgroup for tree rooting. GenBank accession is indicated for each sequence. The country of origin and year of isolation is displayed when available. Circles size denotes the percentage of replicates in the bootstrap analysis (1000 bootstrap analyses). The scale indicates 0.1 substitutions/site. Phylogenetic lineages (Lin.) are indicated according to [5]. Genetic lineage 8 was not included in the tree because only partial sequence, not including NS5, is available (KJ131502).

WNV is a member of the *Flavivirus* genus, within the *Flaviviridae* family. WNV classification was initially based on cross-neutralization reactions, locating it as a member of the Japanese encephalitis virus (JEV) serocomplex. Later on, the molecular phylogeny analyses supported this antigenic classification and revealed the existence of up to nine distinct genetic lineages of WNV (Figure 2), being lineage 1 and 2 the most worldwide distributed [5]. Lineage 2 was restricted to Africa until recently when it was isolated for the first time in Europe from a goshawk in Hungary in 2004 [28]. Since then, lineage 2 strains have been isolated in mosquitoes, humans, and several domestic and wild birds across the continent [29,30,31]. In any case, despite this genomic variability, there is only a single WNV serotype described, which could facilitate the development of unique vaccines to protect against all WNV genotypes. 

### 2.3. Antigenic Structure

**Figure 3:**
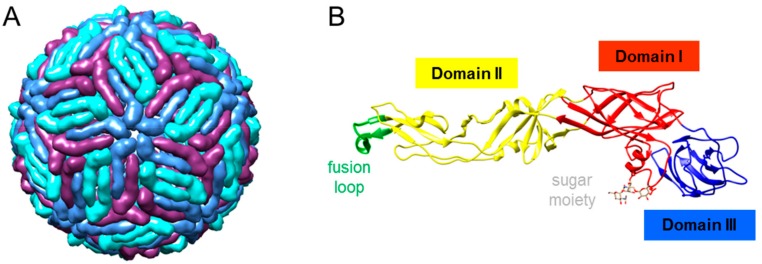
Structure of WNV. (**A**) Cryo-electron microscopy reconstruction of a WNV particle (Protein Data Bank accession 3J0B). E monomers are blue, purple, and turquoise. (**B**) Ribbon diagram of the crystal structure of WNV E glycoprotein (Protein Data Bank accession 2HG0). Domain I is red, domain II is yellow, domain III is blue, and the fusion loop is green. The N-linked sugar moiety of Asn 154 is also displayed. Images were produced using Chimera package [45].

Mature virions are about 50 nm in diameter, and the majority of their surface is occupied by the E glycoprotein (Figure 3A). This external protein shell is composed of 180 copies of E protein arranged as antiparallel homodimers and confers the virions a herringbone T = 3 pseudo-icosahedral symmetry [27]. The E protein is N-glycosylated at Asn 154 in most WNV isolates (Figure 3B). This surface glycoprotein constitutes the major target for neutralizing antibodies, becoming the base of many vaccine candidates [35]. While the lack of glycosylation influences WNV replication in experimentally infected chickens, it does not compromise the induction of antibodies [36]. Notably, the E protein carries both flavivirus cross-reactive and WNV-specific epitopes. The cross-reactivity between WNV and related flaviviruses is the result of the high degree of structural homology between them and can lead to cross-protection but also to adverse effects due to antibody-dependent enhancement of infection [37,38,39,40]. This high cross-reactivity also complicates the precise serological diagnosis of flavivirus infections by immunological techniques, such as ELISA, making necessary the use of confirmatory tests, including related flaviviruses, with the neutralization assay as the gold standard [19]. The E glycoprotein is organized into three domains (DI to DIII), DI is an eight-stranded β-barrel, DII contains the conserved fusion loop (residues 98–110), and DIII adopts an immunoglobulin-like fold form (Figure 3B). Antibody epitopes have been identified in all three domains, with the most prominent neutralizing antibodies targeting DIII, making it an interesting candidate for vaccine development [35]. Antibodies against proteins other than the E have also been identified, so that experimentally infected chickens elicited antibodies against prM and NS1 [41,42]. While antibodies against NS1 have been related to protection in mammals [43], results obtained with red-legged partridges (*Alectoris rufa*) suggest that this could not be always the case [44].

## 3. Avian Susceptibility

Birds are the main vertebrate hosts for WNV, being commonly infected and frequently developing high levels of viremia [6]. Susceptibility of the avian population to the infection can vary depending on the species, being Corvidae (order Passeriformes) the most susceptible family [46,47,48], and important virus amplifiers [7,8,9], that play a key role in the epidemiology of the virus [10,11,12,13,14]. In fact, WNV epidemics in the US were associated with high crow mortality, driving to a significant decrease of native crow species [14,15,16,17]. According to the Centers for Disease Control and Prevention (CDC), birds from almost 300 different species have been found dead since 1999 in the US [49]. This avian WNV-associated mortality has been reported around the world in domestic [50,51,52] and wild birds [11,17,53,54], including endangered species [55,56], as well as in ones adapted to human environments [16].

Differences in pathogenicity, virulence, viremia, the clinical course of the infection, and mortality after experimental infections of birds with WNV strains of either lineage 1 or 2 have been reported [57,58], although no differences have been observed by other authors [8,59].

Main transmission route in birds is by mosquito bites, but other sporadic routes have also been described, such as oral [7,60,61] and bird-to-bird contact [7,13,62,63,64,65], suggesting that WNV-infected birds can be a source of contamination in nature [46,64,66].

A great range of viremias has been reported in different species, which may influence viral transmission. Birds that develop viremia greater than 10^6^ pfu/mL are usually considered competent reservoirs to spread the virus [67], although, for some vectors, it has been described in the range of 10^4^–10^5^ pfu/mL [68]. In fact, while in some species (Columbiformes and Galliformes), viremias are quite low, in others (Passeriformes, Charadriiformes, or Strigiformes) are high, making these species more efficient competent hosts for WNV transmission [7]. Viremia can be detected as soon as one day after infection in high susceptible species [7,69,70,71]. Moreover, WNV has been detected in blood as early as 30–45 min after the bite of infected mosquitoes, suggesting that local replication is not necessary in birds for the primary viremia [72]. Viremia can last up to 7–11 days depending of the avian species [7,59]. Dissemination of the virus to the different tissues has been reported as early as one day after infection in the spleen of crows [73], until 14 days post-infection in kidney and spleen of an American Kestrel (*Falco sparverius*, Falconidae) [7], and even 27 days after infection in the kidney of a horned owl (Strigidae) [74]. WNV can also be detected in oral and fecal swabs from the first day after infection in most of the susceptible species studied with a viral shedding timing that overall reflects that of viremia [8,70,75].

## 4. Avian Pathology

**Figure 4:**
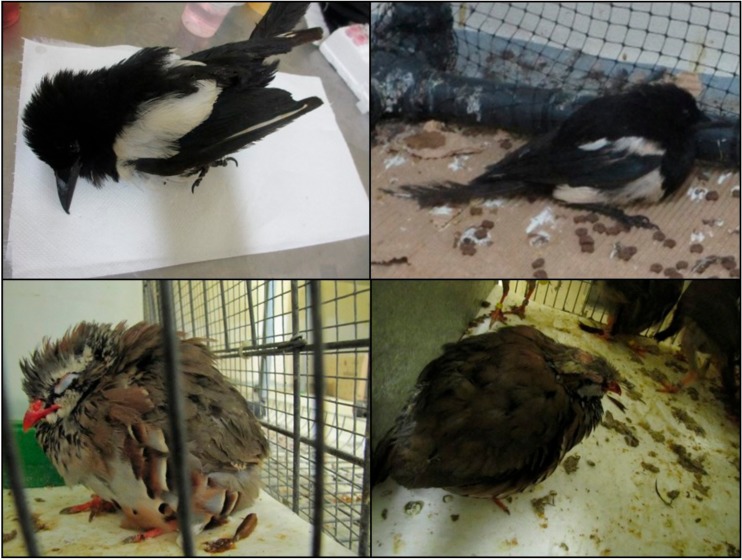
Clinical signs observed in experimentally infected birds. Magpies (upper panel) and partridges (lower panel) experimentally infected with WNV. Birds that die develop high morbidity hours before their death. Evident clinical signs like loss of appetite, ruffled feathers, paralysis, hunchback position, and unresponsiveness are observed in WNV-infected birds from 3 to 8 days post-infection (dpi).

No clinical signs are observed in most WNV-infected birds, and, when they show up, the most common are lethargy, reluctance to move, ruffled feathers, and lack of appetite with marked body weight losses (Figure 4) [7,8,63,75]. Dehydration [70], intermittent head twitching [70], convulsions [47,76], profuse oral and nasal discharge [77], or reduced fecal output [78] are less common. When a fatal outcome occurs, it happens within the first 24 h after the onset of clinical signs [7,8].

Macroscopic lesions are observed in infected birds between 7 and 10 days after infection, although it can be delayed until 21 days post-infection (dpi) [79], and even become chronic [47]. The most affected organs are the brain, presenting encephalitis with cerebellar involvement, heart, liver, spleen, and kidney [75,80,81]. Lesions, such as diffuse pallor or pale foci in epicardium and myocardium [70], or in the hepatic, splenic, and renal parenchyma [80], as well as hepatomegaly and splenomegaly [58,74,75], have also been reported. 

**Figure 5:**
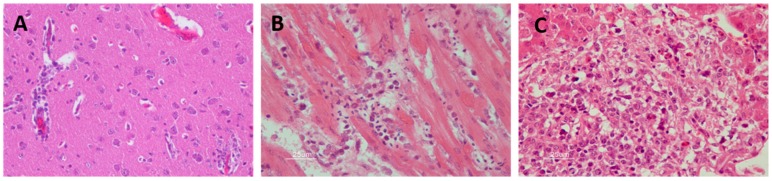
Histopathological findings in WNV-infected red-legged partridge. (**A**) Moderate gliosis, and lymphoplasmacytic and histiocytic perivascular cuffs observed in the brain. (**B**) Extensive myocardial degeneration and necrosis with inflammatory infiltrate composed of lymphocytes, plasma cells, and histiocytes observed in the heart. (**C**) Extensive liver necrosis with predominantly lymphoplasmacytic infiltrate. Images courtesy of Dr. U. Höfle and Dr. V. Gamino.

Among the histopathological findings of the affected organs (Figure 5), central nervous system lesions are mainly characterized by hemorrhages in the brain [81,82,83], mild perivascular cuffs consisting of lymphocytes and plasma cells, scattered individual necrotic neurons, lymphoplasmacytic, and histiocytic meningoencephalitis characterized by gliosis and glial nodules [47,48,59]. The main findings in the heart are lymphoplasmacytic and histiocytic myocarditis with myocardial necrosis, concurrent fibrosis, sometimes with thrombi, hypereosinophilia of cardiomyocytes, myocytolysis, nuclear swelling, pyknosis, loss of striations, myofiber degeneration, and hemorrhages [70,80,84]. Liver lesions include multifocal randomly distributed granulomatous and lymphohistiocytic hepatitis, with mild to moderate coagulative hepatocellular necrosis and deposition of fibrin [75]. The spleen is also affected by WNV infection, where multifocal lymphocytic necrosis occurs characterized by the presence of karyorrhectic nuclear debris [75,81]. Significant histopathological abnormalities present in the kidney are mild multifocal proximal tubular necrosis and mild to moderate lymphoplasmacytic interstitial nephritis that can occasionally be perivascular [70,75,85]. Ocular lesions are also common in WNV-infected birds. These lesions consist of the disarray of the retinal pigmented epithelial cell layer, pectenitis, choroidal or retinal inflammation, cellular necrosis, muscular degeneration in the iris, mild optic neuritis, impaired vision, and even blindness [47,76,86,87,88]. Other less common described lesions include pancreatitis, pulmonary edema, infiltration of lymphocytes, plasma cells and histiocytes in the intestinal tract, necrotizing mucosal duodenitis, myofiber degeneration with lymphoplasmacytic inflammation, and fibrosis in skeletal muscle [89,90].

WNV can persist in the organs of infected birds up to several months [91], thus playing a possible role in viral overwintering and enabling possible new infections through mosquito bites or bird-to-bird transmission [14,92,93].

## 5. Vaccines

Vaccines to protect humans against certain flaviviruses have been available for long time, such as that against yellow fever virus (YFV) in use since more than 80 years, or that against Japanese encephalitis virus (JEV) approved in 2009, and, thus, it is expected that the same principles could be applied to WNV vaccine development. In fact, several commercial formulations are currently available for equid vaccination, and its effectiveness was demonstrated after immunization of horses, which led to a marked decrease of severe WNV disease (WND) in the following years in the US [94,95]. In many cases, experiments with birds have tested some of these commercially available vaccines approved for use in equids [18,19], such as the formalin-inactivated whole-WNV vaccine originally developed by Fort Dodge (Fort Dodge, IA, USA), which has been commercialized under different names (West Nile-Innovator, Duvaxyn^®^ renamed EQUIP WNV^®^) [96,97,98,99,100,101,102,103,104], and was licensed in 2003 and subsequent years (Vetera^®^), a DNA-based vaccine subsequent formulation expressing the prM and E WNV proteins also from Fort Dodge (West Nile-Innovator DNA equine^®^) [97,105], which was licensed in 2004 in the US but later removed from the market in 2010, and a recombinant live canarypox virus vaccine (ALVAC^®^) that expresses the prM/E WNV proteins (Recombitek^®^ Equine West Nile virus in the US, Merial, GA, USA; or Proteq WNV^®^ in Europe) [96,105].

Additionally, experimental prototypes have been assayed, like a chimeric virus based on the yellow fever 17D vaccine strain in which the surface proteins were replaced by that of WNV (ChimeriVax-WN) [106], or a vaccine [64] based on WNV recombinant subviral particles (RSPs) produced by a HeLa-3 cell line stably transfected with a plasmid encoding the signal peptide of the C protein and the prM/E proteins [107]. Several other approaches have used DNA vaccines, like the DNA-plasmid vaccine (pCBWN) [108], also encoding the WNV prM/E proteins [98,105,109,110,111], and a modified version of it [112]. Another DNA vaccine that codes for the prM/M and E proteins of WNV produced by Aldevron [99], and two DNA-plasmid vaccines expressing the ectodomain of the WNV E protein of lineage 1 or 2 in the modified backbone vector pVax1 were also tested [113]. Likewise, a recombinant protein vaccine originally developed for humans, the WN-80E, consisting of a portion of the WNV envelope protein has been assayed too [114]. All these vaccines, commercial and experimental, have been evaluated in different domestic (Table 1) and wild (Table 2) avian species following different routes of administration and vaccination regimens, resulting in varied outcomes.

The availability of vaccines for use in birds, the natural hosts of the virus, will be highly useful, mostly during outbreaks. These vaccines could be used in birds held in captivity in recreational installations and zoos, in wildlife rehabilitation and endangered species breeding centers, and even in birds grown for restocking or hunting activities that are yearly released by the thousands into the environment in many countries. Even more, some of these vaccines could be also administered during surveillance programs [64].

### 5.1. Vaccination in Domestic Birds

The first description of disease and deaths caused by WNV in domestic birds was reported in 1997–1999 in Israel [115], involving hundreds of young geese (*Anser anser*). This species had also been the most affected domestic avian species during virus spread in the US [116]. Symptomatic infections have also been reported in several Psittacine species [90], although experimental infection of birds of these species indicated that they are not very competent [7]. Galliformes, such as chickens (*Gallus gallus domesticus*) and turkeys (*Meleagris gallopavo*), seroconvert but remain asymptomatic. On the other hand, outbreaks among farmed chukar partridges (*Alectoris chukar*) and Impeyan pheasants (*Lophophorus impejanus*) have been reported [52].

After the initial outbreaks in geese in Israel mentioned above [115], both live attenuated and inactivated WNV vaccines have been successfully used there. A live attenuated WNV vaccine was generated by serially passaging a WNV Israeli isolate in a mosquito cell line and selecting an escape mutant using a specific monoclonal antibody [117]. The resulting variant, WN-25A, lost all neuroinvasiveness, while it fully protected geese (20/20) upon a lethal challenge with an Israeli strain isolated from a moribund goose. Later, an attenuated, commercial heterologous flavivirus vaccine derived from Israel turkey meningoencephalitis virus (TMEV) was experimentally tested in laboratory and field settings in geese intra-cranially challenged with WNV two weeks after immunization [115]. The level and duration of protection achieved were quite high and long-lasting (71–93%, 12/17–14/15, protection in laboratory assays, and 39–72%, 9/23–18/25 in the farm ones); however, some goose flocks reacted unfavorably to the vaccination in field trials, showing neurological signs and appreciable mortality. Such undesirable side effects were not observed when they tested a formalin-inactivated WNV strain passaged in suckling mice brains [115]. The same authors reported a 63% (5/8) protection upon intra-cranial challenge when a double dose of this prototype was administered in a single injection and up to 94% (15/16) when a single dose was administered in two injections spaced two weeks apart. Similar studies carried out in farmed goose flocks resulted in 52–80% (13/25–16/20) protection [115]. The efficacy of this vaccine was later evaluated in 829 geese, 298 laboratory-vaccinated, 231 farm-vaccinated, and 300 non-vaccinated, showing 86.58% (258/298), 75.32% (174/231), and 8.33% (25/300) survival rates, respectively, after WNV challenge [118].

Subsequently, an inactivated vaccine was developed using an adapted WNV-Isr98 isolate highly virulent for geese and the PER.C6^®^ cell line platform [119]. When the vaccine was administered with mineral oil as an adjuvant to geese, 91.4% (53/58) survived to the infection, while only 5% (1/20) of the control PER.C6 sham-vaccinated group did. However, the PER.C6-ISR98 candidate did not seem to be sterilizing since, after the challenge, a boost of neutralizing antibodies was detected. In addition, the importance of the use of adjuvants was noted since the administration of the vaccine without adjuvant resulted in 53.3% (32/60) protection [119].

By 2011, the inactivated West Nile-Innovator vaccine was tested for its capability to induce antibodies in chicks and adult thick-billed parrots (*Rhynchopsitta pachyrhyncha*) that received five and three doses with annual boosts along 3 and 7 years, respectively [104]. None of the birds seroconverted after the initial injections, but 2/4 and 3/4 of the chicks developed antibodies 1 and 2 years later, respectively, while only 1/12 and 2/8 of the adults had them 1 and 3 years later, being 6/6 positive after 7 years of annual vaccination. However, as the birds were likely naturally exposed to WNV during the experiment, the interpretation of the results is complicated.

Chimeric vaccines have also been evaluated in domestic birds. So that, an attenuated chimeric vaccine constructed by inserting the prM/E of WNV in dengue virus serotype 4 backbone (WNV/DENV4), and a similar one with a 30-nucleotide deletion in the 3′ non-coding region of DENV4 (WN/DEN4-3′Δ30), which were previously shown to prevent viremia in challenged mice and rhesus macaques [120], were tested in young domestic geese [121]. None of these chimeric vaccines stimulated protective immunity against WNV challenge, and high morbidity rates (3/4 in each group), and a high level of viremia were recorded among vaccinated goose, similar to that in non-vaccinated animals [121].

A different approach was used by testing, in domestic geese, a WNV subunit vaccine that comprised 80% of the E protein (WN-80E) combined with adjuvant and administered twice 4 weeks apart [122]. Using viremia as the clinical endpoint, no virus was detected in the serum of groups of six birds immunized with medium or a high-dose (5 or 10 µg) of the vaccine up to 14 dpi. However, the virus was detected in oral swabs 3–6 dpi in some of the birds, and an increase in antibody titers was observed at 14 dpi, indicating that the vaccine did not induce sterile immunity. Likewise, using a recombinant WNV-E as immunogen to orally (20 µg or 100 µg/dose), or intramuscularly (20 µg/dose), vaccinate Leghorn chickens (*G. gallus domesticus*) three times with a 2-week interval [123], it was shown that, in the birds immunized intramuscularly, the levels of viremia were lower and the total production of WNV E protein-specific IgY was significantly higher than in the animals immunized by the oral route. In this line, a recombinant WNV envelope E (rE) protein produced in insects [124], highly protective in mice [125], was assayed in red-legged partridges. Birds were intramuscularly vaccinated twice at the two-week interval with 10 µg/animal of the rE protein administered with adjuvant, and a control group was similarly sham-immunized. Partridges from both groups were subcutaneously challenged with the NY99 WNV strain [63]. All the rE vaccinated birds (22/22) survived to WNV infection, while 33.3% (6/18) of the sham-immunized partridges succumbed between 3 and 8 dpi, being the mortality rate higher among younger (9 weeks of age at the time of challenge) than among older (13 weeks of age) animals (45.5% vs. 14.3%, respectively). An age-dependent susceptibility had been previously reported in a related species, the chukar partridges, in which 25% mortality was observed in juvenile birds, while no mortality was reported in 14 week-old partridges housed nearby during a WNV outbreak in the US [52], and also in domestic geese [51,126]. Analyses of the humoral immune response elicited by rE vaccinated partridges showed that all animals were ELISA positive after two immunizations [63], similar to what had previously been described in geese and American crows (*Corvus brachyrhynchos*) [98,119]. Again, the immunity elicited by rE vaccinated partridges was not sterilizing, as viremia was detected in 4/22 vaccinated birds 3 dpi, and antibody titers significantly increased after viral challenge to levels similar to those found in non-vaccinated animals. Similar results had been observed after vaccination of geese, red-tailed hawks, and Western scrub-jays (*Aphelocoma californica*) [105,110,119].

Another study tested five different vaccine candidates administered intramuscularly in 47 geese [116]. The prototypes tested were an inactivated whole virus prepared with mineral oil as an adjuvant, three recombinant viruses containing the WNV prM/E (the canarypox viruses ALVAC vCP2017 and vCP2018; and the fowlpox virus vFP2000), and an exogenously produced WNV E protein. Birds were challenged 2 weeks after the booster immunization, except in the last case that was done after 1 week. Since no geese died in the challenged sham-immunized groups and only one developed clinical signs, protection was measured through the following five parameters: clinical pathogenicity index (CPI), plasma virus-positive geese on days 1–4 post-inoculation, plasma virus titers, brain histological lesion rates, and severity scores. The best protection was achieved with the vFP2000 fowlpox virus, which was the one that best scored in the five parameters, followed by the vCP2018 canarypox virus that did it in four, the vCP2017 in three, the E protein in one, and the oil-emulsion WNV in none. 

### 5.2. Vaccination in Wild Birds

Vaccination of wild species presents associated problems, such as the limited access to individuals, aggravated by the high number of susceptible species described, and environmental safety concerns, especially with attenuated or genetically engineered live virus-based vaccines. However, since, in many of them, WNV-related pathogenesis is not clinically relevant and/or they have a limited, if any, role in virus ecology, most of them do not seem to represent a target for vaccine campaigns implementation. Indeed, most efforts in experimental vaccine development have selected members of the Corvidae family as model, although raptors (Accipitridae and Falconidae), nocturnal bird preys (Strigidae and Tytonidae), and members of other families (Phoenicopteridae, Spheniscidae, Gruidae, Turdidae, Cathartidae, Phasianidae, and Anatidae) have also been used (Table 2). For most of them, WNV-associated mortality has been described [8,74,128], and some have been related to virus spreading and re-introduction in different geographical areas due to their migratory behavior.

The main aim of a vaccine is conferring protection. A single intramuscular dose of the pCBWN DNA vaccine administered to fish crows resulted in 100% (8/8) survival rate in comparison with the 50% recorded in non-vaccinated (5/10), or orally immunized (4/8) birds [111]. However, the same vaccine intramuscularly administered to American crows resulted in 44% (4/9) survival, while none (0/10) of the sham-inoculated birds survived [98]. The same authors reported up to 60% (6/10) increased survival rate when intramuscular immunization was performed with an adjuvant, a lack of protection with the adjuvant alone (0/8), or when the oral route was used (0/10), and a low one (11%, 1/9) when the West Nile-Innovator vaccine was intramuscularly administered [98]. Another study reported that a single intramuscular vaccination of Western scrub-jays with the pCBWN vaccine protected 100% (3/3) of the birds, and that 80% (4/5) of the corvids intramuscularly vaccinated with a single dose of the Fort Dodge West Nile-Innovator DNA equine^®^ vaccine or the Recombitek^®^ Equine West Nile virus formulation also survived to the infection compared to the 40% (2/5) of survival reached by the control group [105]. Dispensation to falcons of the Duvaxyn^®^ inactivated vaccine resulted in 80% (4/5) and 100% (4/4) protection when administered twice or thrice, respectively, and 100% protection with two (5/5) or three doses (4/4) was achieved with the Recombitek^®^ Equine West Nile virus formulation, while only 50% (4/8) survival was recorded in non-vaccinated animals [96]. Two DNA vaccines that express the ectodomain of the E protein of WNV of lineage 1 or 2 also tested in large falcons conferred protection against a WNV challenge and, based on their results with different protocols, the authors indicated that protection was dependent on the lineage, regimen, and way of administration used. Birds intramuscularly immunized with the plasmid, encoding the protein of lineage 2, reached 100% (5/5) survival in comparison to the 60% (3/5) reached by those immunized with that of lineage 1, or the 50% (4/8) showed by non-vaccinated birds [113]. Finally, a single intramuscular dose of an RSP-based vaccine protected magpies (*Pica pica*) as 71.4% (5/7) of the vaccinated birds survived to viral challenge, compared to the 22.2% (2/9) survival rate observed among sham-immunized magpies [64].

Remarkably, in one study [106], vaccination of fish crows with the chimeric ChimeriVax-WN resulted in a high mortality rate upon WNV challenge when compared with non-vaccinated birds (25%, 2/8 and 0%, 0/4, respectively) that was accused to a possible antibody-dependent viral enhancement effect, although such effect has not been observed in nature.

Reduced morbidity and pathogenicity were also observed upon experimental vaccination of wild birds. Thus, a reduced pathology was noted in sandhill cranes (*Grus Canadensis*) vaccinated with the Fort Dodge inactivated vaccine [103], clinical signs alleviations were also reported after administration of three doses of Duvaxyn^®^ or Recombitek^®^ Equine West Nile virus vaccines in large falcons [96], and reduced clinical scores and antigen deposition in their organs upon DNA vaccination were also documented [113]. Likewise, a less pronounced and shorter loss of weight and a lack of clinical signs were described in RSP-vaccinated magpies that survived to viral challenge [64].

### 5.3. Sterilizing Immunity

As commented above, most of the tested vaccines conferred protection when animals were challenged, but, in none of them, induction of sterilizing immunity was observed, as viral replication could be detected in vaccinated birds, although with the exception of one study performed in vaccinated Western scrub-jays in which no reduction of viral titers was reported [105], those were usually lower in vaccinated than in sham-immunized animals [64,96,98,103,109,110,111,113]. For instance, viremia was detected in 4/22 rE vaccinated partridges 3 dpi, and antibody titers increased significantly after viral challenge to levels similar to those found in non-vaccinated animals [63]. 

On the other hand, in many cases [64,96,103,109,113], viremia reached levels below what is considered necessary to be a competent reservoir [7], although, for some vaccines, two [113] or three [96,103] doses were required to achieve it. In this respect, it was reported that for *Culex pipiens* and *Culex quinquefasciatus* [129], two vectors considered key to virus maintenance, these levels must be above 10^5^ pfu/mL, although lower viremia levels (around 10^4^ pfu/mL) also seemed to be relevant for other vectors, such as *Culex univittatus* or *Culex perexiguus* [68]. Additionally, a boost of antibodies in vaccinated birds was usually observed after viral challenge [64,103,105,110].

### 5.4. Immunogenicity

Induction of antibodies prior to challenge has not always been detected [101,103,109] and, if so, they are present at low levels [96,100,105,110,112,113], and not in all vaccinated individuals [64,97,98,99,100,102,106,110,111,113]. As commented above, humoral immune response priming that induces an enhanced and prominent/lasting antibody production has been described after viral challenge in vaccinated birds [64,103,105,110]. Although an association between antibody induction and protection has been suggested [64,98], sometimes, as no challenge was conducted, this hypothesis could not be confirmed [97,99,100,101,102,112,114]. For instance, a non-replicating recombinant adenoviruses, expressing either the WNV envelope (rAdE) or the NS3 (rAdNS3) proteins, was assayed in Japanese quails (*Coturnix japonica*) [127], but, since no challenge was performed, the efficacy of the vaccines was measured in terms of WNV-specific antibodies levels and T cells specific activation, which were both increased in vaccinated birds compared to unvaccinated controls. This antibody response was higher and more robust with the rAdE candidate than with the rAdNS3, probably due to the expression of the entire E protein on the cell surface, thus allowing B cells to bind to any available epitope on the molecule. Even more, vaccination with rAdE triggered the activation of more WNV-specific CD4^+^ T cells, which would be required to fully activate the WNV primed B cells to produce antibodies. In line with this, vaccines based on DNA and live vectors favor the availability of cytotoxic T lymphocytes (CTLs) epitopes and therefore, should improve protection after viral challenge if T-cell responses are important. One work performed in large falcons compared the efficacy of the inactivated Duvaxyn^®^ and the live vector-based Recombitek^®^ Equine West Nile virus vaccines, showing a slightly better protection of the later one, as mentioned above [96], and, thus, pointing to a protective role of the cellular immune response. However, another study conducted in American crows with the pCBWN and the inactivated West Nile-Innovator vaccines showed the opposite results, as survival rates were 44% (4/9) and 11% (1/9), respectively [98]. Moreover, many vaccines tested included adjuvant, which could favor antigen presentation to T cells and prolong the presence of viral antigens for B cell recognition. So that, the pCBWN vaccine provided microencapsulated in sodium alginate [111], or with aluminum [98,110], increased the survival rates of immunized and challenged American crows when compared with those which received the vaccine alone (44%, 4/9 and 60%, 6/10, respectively) [98]. Aluminum has also been used with DNA [99] and RSP-based vaccines with good results [64].

### 5.5. Herd Immunity

Horizontal transmission of WNV in experimentally infected birds was early described [7]. This can be due to direct contact or by fecal-oral route since the virus can be detected in cloacal and oral swabs [7,8,61,128] and in feathers of infected birds [8]. Moreover, viremia levels reached in the absence of mosquito-borne transmission can be as high as those obtained by mosquito natural exposure [7]. Several vaccines have demonstrated to be effective in diminishing the risk of this type of transmission as they were able to either shorten [103,110] or reduce viral shedding [96,110,113] and virus presence in feathers [8]. Even more, it has been reported that RSPs vaccination completely broke horizontal transmission, as none (0/4) of the contact birds housed with challenged-vaccinated magpies got infected in contrast to 50% (3/6) that did it when were housed with challenged, unvaccinated cage-mates [64]. These data point to the induction of herd immunity through bird vaccination. 

On the other hand, transmission in the absence of mosquito-borne infection has also been reported by the consumption of birds infected with WNV by scavenger species [60,128,130]. Therefore, and even in the absence of sterilizing immunity, reduction of viral load in organs after vaccination is desirable. In fact, reduction of viral load in the organs of challenged falcons vaccinated with commercially available WNV vaccines, such as Duvaxyn^®^, Recombitek^®^ Equine West Nile virus [96], or with DNA-plasmid vaccines encoding the ectodomain of the E protein, has been reported [113].

### 5.6. Side Effects and Feasibility

An additional important point that must be taken into account for vaccine implementation is the lack of undesirable side effects. Even though local inflammation at the site of administration has been observed, probably due to hypersensitivity reactions to the vaccines or the natural effect of the adjuvants employed in some DNA-based and RSP-based vaccines [64,113], in most cases, no such side effects have been described. Two commercial Fort Dodge vaccines designed for equids (virus inactivated and DNA) showed no adverse side effects in corvids, cranes, or falcons [96,103,105]. The inactivated formulation has also been assayed in flamingos, hawks, eagles, vultures, owls, penguins, and wild chickens [99,100,101,102], showing good safety profiles, although, since no viral challenge was done, the immunopathological effects related to the vaccine during the infection were not evaluated. Moreover, vaccines based on live vectors can provoke adverse effects, such as the unexpected increase in mortality observed in corvids vaccinated with the ChimeriVax-WN [106], the development of necrotic lesions also in corvids [105], the massive local inflammation reported in falcons immunized with the Recombitek^®^ Equine West Nile virus formulation [96], or the neurological signs and appreciable mortality observed in geese vaccinated with the heterologous TMEV-based vaccine candidate [115]. Even more, vaccines should avoid any environmental effects. In this regard, shedding by the fecal-oral route of vaccines based on virus or plasmid was not found in falcons immunized with Recombitek^®^ Equine West Nile virus [96], or with DNA-plasmids vaccines expressing WNV proteins of lineage 1 or 2 [113].

As commented above, the biggest drawback for implementation feasibility of wild bird vaccines is access to the target host. This could be bypassed if herd immunity can be established, preferably by oral vaccination in, for example, feeding stations, which has already been useful for controlling other zoonotic diseases, such as rabies [131]. However, so far, experimental vaccination of birds by the oral route has failed in conferring protection [98,111], and it has not even able to stimulate the production of antibodies. In any case, avian vaccination can be a realistic option in specific situations, such as in birds grown for restocking activities, endangered species in captive breeding projects, wildlife reserves, recreation installations, or during epidemiological surveillance programs.

## 6. Conclusions

The objective of any vaccine is the induction of protection that, preferably, should be long-lasting and sterilizing, and induce herd immunity. Experimental vaccination with different formulations (attenuated, inactivated, recombinant viruses, and subunits and DNA-based candidates) has been assayed in domestic and wild birds from different species and ages following different routes of administration and regimens, which has resulted in varied outcomes. Even though, due to logistical and ethical concerns, among others, the number of birds included in the studies has generally been rather low, mainly when wild birds were used, the reported data indicate that, overall, vaccination induces humoral and, more probably, cellular responses, and reduces WNV-associated disease, lesions, viremia, viral shedding, and, more significantly, mortality. However, no sterilizing immunity has been observed, induction of antibodies has not always been recorded, and, if detected, it was not always in every bird. Remarkably, when evaluated, no horizontal transmission from challenged-vaccinated birds has been observed, pointing to the induction of herd immunity that would prevent virus maintenance in the environment and, thus, its spread. Nevertheless, the implementation of bird vaccines faces several drawbacks, such as the difficult feasibility of access to the target host, mainly for wild species, as well as the administration route, as oral, the most feasible one, has failed to confer protection. In any case, the availability of effective avian vaccines against WNV would be very helpful, mainly during outbreaks, and therefore, research should go on.

## Figures and Tables

**Table 1:** Vaccines tested in domestic birds.

Vaccines					Birds			Results				Ref.
Type	Name	Route	Dose	Adjuvant	Family	Latin Name	Common Name	Safety Concerns	Protection	Competence Break	Antibodies	
Live Recombinant Vector	vCP2017	IM	×2	PM	Anatidae	*Anser anser*	Domestic geese	NC	NA	yes	yes (NAI)	[116]
	vCP2018	IM	×2	PM	Anatidae	*Anser anser*	Domestic geese	NC	NA	yes	yes (NAI)	
	vFP2000	IM	×2	PM	Anatidae	*Anser anser*	Domestic geese	NC	NA	yes	yes	
	rAdE	IM	×2	no	Phasianidae	*Coturnix japonica*	Japanese quail	NC	NT	NA	yes	[127]
	rAdNS3	IM	×2	no	Phasianidae	*Coturnix japonica*	Japanese quail	NC	NT	NA	yes (L)	
Live Attenuated Whole Virus	WNI-25A	IP	×1	no	Anatidae	*Anser anser*	Domestic geese	NC	100% vs. 0%	NT	NT	[117]
	TMEV	SC	NC	no	Anatidae	*Anser anser*	Domestic geese	yes	71–93% vs. 0%	NT	NT	[115]
		IM	NC	no	Anatidae	*Anser anser*	Domestic geese	yes	82–93% vs. 0%	NT	NT	
Inactivated Whole Virus	TME-formalin boosted with live TME	SC	NC	oil	Anatidae	*Anser anser*	Domestic geese	no	100% vs. 0%	NT	NT	[115]
		IM	NC	oil	Anatidae	*Anser anser*	Domestic geese	no	100% vs. 0%	NT	NT	
	WNV-formalin inactivated	SC	×2	mineral oil	Anatidae	*Anser anser*	Domestic geese	no	92% vs. 0%	NT	NT	[115]
		IM	×2	mineral oil	Anatidae	*Anser anser*	Domestic geese	no	80% vs. 0%	NT	NT	
		SC	×2	mineral oil	Anatidae	*Anser anser*	Domestic geese	no	75–86% vs. 8%	NT	NT	[118]
	PER.C6-ISR98	SC	×2	mineral oil	Anatidae	*Anser anser*	Domestic geese	no	91.4% vs. *5*%	NT	yes (L)	
	West Nile - Innovator	IM	×3*	MetaStim	Psittacidae	*Rhynchopsitta pachyrhyncha*	Thick-billed parrots	NC	NT	NA	yes**	[104]
	WNV- formaldehyde inactivated	IM	×2	mineral oil	Anatidae	*Anser anser*	Domestic geese	NC	NA	no	yes (NAI)	[116]
Recombinant Protein	WN-80E	IM	×2	ISA 720	Anatidae	*Anser anser*	Domestic geese	no	NA	NA	yes (L)	[122]
		IM	×2	no	Anatidae	*Anser anser*	Domestic geese	no	NA	NA	no	
	rE protein	OR	×3	LTK63	Phasianidae	*Gallus gallus*	Domestic chickens	NC	NA	NA	no	[123]
		IM	×3	LTK63	Phasianidae	*Gallus gallus*	Domestic chickens	NC	NA	NA	yes	
	E protein	IM	×2	mineral oil	Anatidae	*Anser anser*	Domestic geese	NC	NA	NA	yes (NAI)	[116]
	rE protein	IM	×3	Specol	Phasianidae	*Alectoris rufa*	Red-legged partridges	no	100% vs. 33%	NA	yes	[63]

* Vaccine administered x3 in the first year followed by yearly boosters (7 years); ** all animal seroconverted 3 (chicks) to 7 (adults) years after annuall boosters; TMEV: Israel turkey meningoencephalitis virus; WNV: West Nile virus; SC: subcutaneous; IM: intramuscular; IP: intraperitoneal; OR: oral; PM: adjuvant provided by the manufacturer; NC: not commented; NA: not applicable; NT: not tested; NAI: not in all individuals; L: low titer; Break of competence: vaccine lowers viremia levels below the threshold of competence (see text).

**Table 2:** Vaccines tested in wild birds.

Vaccines					Birds Used			Results				Ref.
Type	Name	Route	Dose	Adjuvant	Family	Latin Name	Common Name	Safety Concerns	Protection	Competence Break	Antibodies	
Chimeric Virus	ChimeriVax- WN	SC	×3	no	Corvidae	*Corvus ossifragus*	Fish crows	yes	NA	no	yes (NAI, L)	[106]
Live Recombinant Vector	Recombitek^®^ Equine West Nile Virus	IM	×1	no	Corvidae	*Aphelocoma californica*	Western scrub-jays	yes	80% vs. 40%	no	yes (L)	[105]
		IM	×2/×3	no	Falconidae	Falco *spp*	Large falcons	yes	100% vs. 50%	yes	yes (L) 2× yes (NAI) 3×	[96]
Inactivated Whole Virus	West Nile - Innovator	IM	×1	MetaStim	Phoenicopteridae	*Phoenicopterus chilensis*	Chilean flamingos	no	NT	NA	no	[101]
		IM	×1	MetaStim	Accipitridae	*Buteo jamaicensis*	Red-tailed hawks	no	NT	NA	no	
		IM	×3	MetaStim	Accipitridae	*Buteo jamaicensis*	Red-tailed hawks	NR	NT	NA	yes*	[100]
		IM	×3	MetaStim	Accipitridae	*Parabuteo unicinctus*	Harris’ hawks					
		IM	×3	MetaStim	Accipitridae	*Accipiter cooperii*	Cooper’s hawks					
		IM	×3	MetaStim	Accipitridae	*Buteo swainsoni*	Swainson’s hawks					
		IM	×3	MetaStim	Accipitridae	*Haliaeetus leucocephalus*	Bald eagle					
		IM	×3	MetaStim	Accipitridae	*Aquila chrysaetos*	Golden eagle					
		IM	×3	MetaStim	Falconidae	*Falco mexicanus*	Prairie falcon					
		IM	×3	MetaStim	Falconidae	*Falco peregrinus*	Peregrine falcon					
		IM	×3	MetaStim	Corvidae	*Corvus corax*	Common raven					
		IM	×3	MetaStim	Cathartidae	*Cathartes aura*	Turkey vultures					
		IM	×3	MetaStim	Strigidae	*Otus kennicottii*	Western screech- owls					
		IM	×3	MetaStim	Strigidae	*Bubo virginianus*	Great horned owls					
		IM	×3	MetaStim	Tytonidae	*Athene cunicularia*	Burrowing owls					
		IM	×3	MetaStim	Tytonidae	*Tyto alba*	Barn owls					
		IM	×2	MetaStim	Corvidae	*Corvus brachyrhynchos*	American crow	NC	11% vs. 0%	no	yes (NAI, L)	[98]
		IM	×2	MetaStim	Spheniscidae	*Spheniscus demersus*	Black-footed penguins	no	NT	NA	yes (NAI)	[102]
		IM	×2	MetaStim	Spheniscidae	*Eudyptula minor*	Little blue penguins	no	NT	NA	yes (NAI)	
		IM	×3	MetaStim	Phasianidae	*Tympanuchus cupido attwateri*	Attwater’s prairie chickens	no	NT	NA	yes (NAI)	
		IM	×3	MetaStim	Phoenicopteridae	*Phoenicopterus chilensis*	Chilean flamingos	no	NT	NA	yes (NAI)	
		IM	×2	MetaStim	Phoenicopteridae	*Phoenicopterus ruber*	American flamingos	no	NT	NA	yes (NAI)	
		IM	×2	MetaStim	Spheniscidae	*Spheniscus humboldti*	Humboldt penguins	no	NT	NA	yes (NAI)	[99]
		IM	×2	MetaStim	Spheniscidae	*Spheniscus magellanicus*	Magellanic penguins	no	NT	NA	yes (NAI)	
		IM	×2	MetaStim	Spheniscidae	*Pygoscelis papua*	Gentoo penguins	no	NT	NA	yes (NAI)	
		IM	×2	MetaStim	Spheniscidae	*Eudyptes chrysocome*	Rockhopper penguins	no	NT	NA	yes (NAI)	
		IM	×3	MetaStim	Gruidae	*Grus canadensis*	Sandhill cranes	no	NA	yes	no	[103]
		IM	×3**	MetaStim	Corvidae	*Aphelocoma insularis*	Island scrub-jays	no	NT	NA	yes (NAI)	[97]
		IM	×2/×3	MetaStim	Falconidae	Falco *spp*	Large falcons	NR	80% (x2)–100% (x3) vs. 50%	no (2x) yes (3x)	yes	[96]***
Recombinant Subunit	RSP-WNV	SC	×1	aluminum hydroxide	Corvidae	*Pica pica*	Eurasian magpies	NR	71% vs. 22%	yes	yes (NAI)	[64]
DNA	West Nile-Innovator DNA equine^®^	IM	×1	MetaStim	Corvidae	*Aphelocoma insularis*	Island scrub-jays	no	NT	NA	yes (NAI)	[97]
		IM	×1	MetaStim	Corvidae	*Aphelocoma californica*	Western scrub-jays	NR	80% vs. 40%	no	yes (L)	[105]
	pCBWN	OR	×1	sodium alginate	Corvidae	*Corvus ossifragus*	Fish crows	NC	100% vs. 50%	NA	yes (NAI)	[111]
		IM	×1	sodium alginate	Corvidae	*Corvus ossifragus*	Fish crows	NC	no	NA	no	
		IM	×2	no	Corvidae	*Corvus brachyrhynchos*	American crows	NC	44% vs. 0%	no	yes (NAI, L)	[98]
		IM	×2	aluminum phosphate	Corvidae	*Corvus brachyrhynchos*	American crows	NC	60% vs. 0%	no	yes (NAI, L)	
		OR	×4	no	Corvidae	*Corvus brachyrhynchos*	American crows	NC	no	no	no	
		IM	×1	no	Turdidae	*Turdus migratorius*	American robins	NC	NA	yes	no	[109]
		IM	×2	aluminum phosphate	Accipitridae	*Buteo jamaicensis*	Red-tailed hawks	no	NA	NA	yes (NAI, L)	[110]
		IM	×1	no	Corvidae	*Aphelocoma californica*	Western scrub-jays	NR	100% vs. 40%	no	yes (L)	[105]
	pCBWN-Amp C	IM	×2	aluminum phosphate	Cathartidae	*Vultur gryphus*	Andean condors	no	NT	NA	yes (L)	[112]
		IM	×2	aluminum phosphate	Cathartidae	*Gymnogyps californianus*	California condors	no	NT	NA	yes (L)	
	prM/E- Aldevron	IM	×2	aluminum hydroxide	Spheniscidae	*Spheniscus humboldti*	Humboldt penguins	no	NT	NA	yes (NAI)	[99]
		IM	×2	aluminum hydroxide	Spheniscidae	*Spheniscus magellanicus*	Magellanic penguins	no	NT	NA	yes (NAI)	
		IM	×2	aluminum hydroxide	Spheniscidae	*Pygoscelis papua*	Gentoo penguins	no	NT	NA	yes (NAI)	
		IM	×2	aluminum hydroxide	Spheniscidae	*Eudyptes chrysocome*	Rockhopper penguins	no	NT	NA	yes (NAI)	
	pVax-E-ect-lin.1	IM	×2	no	Falconidae	Falco *spp*	Large falcons	NR	60% vs. 50%	yes	yes (NAI, L)	[113]
	pVax-E-ect-lin.2	IM	×2	no	Falconidae	Falco *spp*	Large falcons	NR	100% vs. 50%	yes	yes (NAI, L)	
	pVax-E-ect-lin.1	IM+EP	×2	no	Falconidae	Falco *spp*	Large falcons	NR	100% vs. 50%	yes	yes (NAI, L)	
	pVax-E-ect-lin.1/rE-dIII	IM-EP/ SC	×1/×2	no/ISA 70	Falconidae	Falco *spp*	Large falcons	NR	80% vs. 50%	yes	yes (NAI, L)	
Recombinant Protein	WN-80E	IM	×2	ISA 720	Anatidae	*Branta sandvicensis*	Hawaiian Nēnē	no	NT	NA	yes	[114]

* Low number of single species in each group (few seroconversion and low titers with the highest dose tested); ** Whenever possible, as birds were captured and release after each vaccination and eventually recaptured; *** Duvaxyn^®^ commercial formulation used; RSP: recombinant subviral particles; WNV: West Nile virus; SC: subcutaneous; IM: intramuscular; OR: oral; IM+EP: intramuscular + electroporation; NC: not commented; NR: not relevant; NA: not applicable; NT: not tested; NAI: not in all individuals; L: low titer; Break of competence: vaccine lowers viremia levels below the threshold of competence (see text).
